# Tenuigenin protects dopaminergic neurons from inflammation via suppressing NLRP3 inflammasome activation in microglia

**DOI:** 10.1186/s12974-017-1036-x

**Published:** 2017-12-20

**Authors:** Zheng Fan, Zhigang Liang, Hui Yang, Yuting Pan, Yan Zheng, Xiaomin Wang

**Affiliations:** 1 Department of Pharmacology, School of Basic Medical Sciences, Capital Medical University, Beijing, 100069 People’s Republic of China; 2 Department of Neurology, Yantai Yuhuangding Hospital Affiliated to Qingdao University, Yantai, 264000 Shandong People’s Republic of China; 3 Core Facility Center, Capital Medical University, Beijing, People’s Republic of China; 4 Department of Physiology, School of Basic Medical Sciences, Capital Medical University, Beijing, People’s Republic of China; 5 Department of Neurobiology, School of Basic Medical Sciences, Capital Medical University, Beijing, People’s Republic of China; 6 Beijing Institute for Brain Disorders, Beijing, People’s Republic of China

**Keywords:** Parkinson’s disease, Tenuigenin, NLRP3 inflammasome, Microglia, Inflammation

## Abstract

**Background:**

Emerging evidence indicates that nod-like receptor family, pyrin domain-containing 3 (NLRP3) inflammasome-induced inflammation plays a crucial role in the pathogenesis of Parkinson’s disease (PD). Thus, inhibition of NLRP3 inflammasome activation may offer a therapeutic benefit in the treatment of PD. Tenuigenin, a major active component of *Polygala tenuifolia*, has been shown to have potential anti-inflammatory activity, but the underlying mechanisms remain obscure.

**Methods:**

In the present study, the 1-methyl-4-phenyl-1,2,3,6-tetrahydropyridine (MPTP)-induced mouse model of PD was established to explore the effect of tenuigenin on dopaminergic neurons in substantia nigra. We next activated NLRP3 inflammasome in both BV2 microglia cells and adult mice to investigate the mechanisms for the neuroprotective effect of tenuigenin.

**Results:**

We demonstrated that treatment with tenuigenin increased striatal dopaminergic levels and improved motor impairment induced by MPTP. Also, tenuigenin significantly ameliorated the degeneration of dopaminergic neurons and inhibited NLRP3 inflammasome activation in substantia nigra of MPTP mouse model. We further found that tenuigenin reduced intracellular reactive oxygen species (ROS) production and suppressed NLRP3 inflammasome activation, subsequent caspase-1 cleavage, and interleukin-1β secretion in BV2 microglia cells. These data indicate that tenuigenin inhibits the activation of NLRP3 inflammasome via downregulating ROS. Correspondingly, in vivo data showed that tenuigenin attenuates microglia activation induced by lipopolysaccharide (LPS) in substantia nigra via suppressing NLRP3 inflammasome.

**Conclusions:**

Our findings reveal that tenuigenin protects dopaminergic neurons from inflammation partly through inhibition of NLRP3 inflammasome activation in microglia, and suggest the promising clinical use of tenuigenin for PD therapy.

## Background

Parkinson’s disease (PD) is one of the most common neurodegenerative disorders, characterized by a progressive loss of dopaminergic neurons in substantia nigra compacta (SNc) and depletion of dopamine in the striatum, leading to debilitating problems with resting tremor, rigidity, bradykinesia, and gait disturbance [1]. While the pathogenic mechanisms that ultimately cause PD are still unclear, it is believed that the progressive nature of PD is characterized by chronic inflammation-induced dopaminergic neuronal degeneration. The hallmarks of neuroinflammation are the presence of activated microglia in the brain and increased production of chemokines, cytokines, and neurotoxic proteins. It has been demonstrated that in the brains of PD patients, levels of pro-inflammatory mediators, including tumor necrosis factor-α (TNF-α), interleukin-1β (IL-1β), IL-6, and ROS are elevated [2]. A meta-analysis of anti-inflammatory drug trials revealed an association between nonsteroidal anti-inflammatory drug (NSAID) use and reduced risk for developing PD possibly implicating neuroinflammatory processes in the disease [3].

Among these pro-inflammatory cytokines, IL-1β has been recognized to be essential for initiation and progress of PD. Enhanced expression of IL-1β has been observed both in the brain and in the periphery of PD patients as well as animal models [4, 5]. The matured IL-1β is tightly controlled by cytosolic multiprotein complexes called “inflammasomes,” which recognize a large number of stimuli, such as danger-associated molecular patterns (DAMPs) and pathogen-associated molecular patterns (PAMPs). The NLRP3 inflammasome is highly expressed in microglia and essential to the process of neuroinflammation [6, 7]. It composed of nod-like receptor protein NLRP3, adaptor protein ASC, and pro-caspase-1, then activated by lots of stimuli, including bacterial, fungal, and viral components, and endogenous danger molecules such as extracellular adenosine 5′-triphosphate (ATP), uric acid crystals, silica crystals, and amyloid-β. The activation of NLRP3 inflammasome promotes the maturation and release of IL-1β, so it plays critical roles in the initiation of inflammation [8, 9]. In our previous studies, we had reported the NLRP3 inflammasome involved in the pathogenesis of PD and might be a potential target for PD therapy [10, 11].

Tenuigenin (TEN) is a natural extract from *Polygala tenuifolia* root, a traditional Chinese herb that has been widely prescribed in traditional Chinese medicine for treating amnesia, neurasthenia, insomnia, palpitation, and cognitive dysfunction for thousands of years. It had reported tenuigenin possesses various pharmacological activities for anti-oxidant, anti-aging, and anti-inflammatory. For example, in vitro, tenuigenin inhibited LPS-triggered inflammatory cytokine production including prostaglandin E_2_ (PGE_2_), cyclooxygenase-2 (COX-2), and inducible nitric oxide synthase (iNOS) in macrophages [12]. Furthermore, tenuigenin exhibited protective effects against LPS-induced acute kidney injury in mice [13]. Notably, our previous studies demonstrated that tenuigenin protected dopaminergic neurons from inflammation induced by intraventricular injection of LPS in rats [14]. Tenuigenin also protected SH-SY5Y cells from 6-hydroxydopamine (6-OHDA)-induced damage [15]. These results indicate that tenuigenin exerts neuroprotection in the progression of PD. Although the anti-inflammatory effect of tenuigenin and its implication in the pathology of PD are emerging, the mechanisms are still poorly understood.

In the present study, we prepared a classic systemic PD model based on the administration of MPTP, which has selective toxicity for dopaminergic neurons. Then, we explored the effects of tenuigenin on motor behavior, dopamine content, dopaminergic neuronal degeneration, and NLRP3 inflammasome activation. Furthermore, we activated NLRP3 inflammasome in both BV2 microglia cells and adult mice to clarify the anti-inflammatory effect of tenuigenin. Our study demonstrates that tenuigenin protects dopaminergic neurons in the substantia nigra of PD mice via suppressing NLRP3 inflammasome activation in microglia, suggesting that tenuigenin may be a promising drug for PD therapy.

## Methods

### Antibodies and reagents

**Fig. 1:**
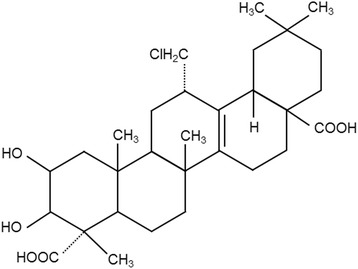
Chemical structure of tenuigenin

Tenuigenin (molecular formula: C_30_H_45_ClO_6_; average molecular weight: 537.14 kDa, chemical structure showed in Fig. 1) was purchased from the Chinese National Institute for the Control of Pharmaceutical and Biological Products (111572-200702) with a purity of 98.7%. LPS (*Escherichia coli* 0111:B4, L4391), MPTP (M0896), ATP (A2385), and MSU (U2875) were purchased from Sigma-Aldrich (St. Louis, MO, USA). Mouse IL-1β ELISA Kits were purchased from R&D Systems. ROS-specific fluorescent probe cell-permeant 2′,7′-dichlorodihydrofluorescein diacetate (H2DCFDA) was purchased from Invitrogen (Thermo Fisher Scientific Inc., USA). The antibodies used in this study are the following: rabbit monoclonal anti-NLRP3 (1:1000, Cell Signaling Technology, Beverly, MA, USA), rabbit anti-caspase-1 (1:800, Santa Cruz Biotechnology, USA), goat anti-IL-1β (1:800, R&D Systems, Minneapolis, USA), mouse monoclonal anti-β-actin (1:5000, Sigma-Aldrich), mouse monoclonal anti-tyrosine hydroxylase (TH, 1:1000, Sigma-Aldrich), and rabbit polyclonal anti-ionized calcium-binding adaptor molecule 1 (Iba-1, 1:500, Wako, Osaka, Japan). The reagents obtained from other sources are detailed throughout the following text.

### Animal models and drug administration

Animals were maintained under specific pathogen-free conditions and treated according to the protocols approved by IACUC (Institutional Animal Care and Use Committee of Capital Medical University).

MPTP acute model: Male C57BL/6J mice (12-week) were randomly divided into five groups: saline, MPTP, and MPTP plus tenuigenin (low dose, 25 mg/kg), MPTP plus tenuigenin (high dose, 50 mg/kg), and tenuigenin (high dose, 50 mg/kg) alone. Mice were pre-treatment daily with tenuigenin for 10 days and then given MPTP (20 mg/kg) intraperitoneally four times at 2-h interval after tenuigenin administration. The same volume of saline was injected in the vehicle group. Two days after the last injection, behavioral assessments were performed using the open field test and rotarod test. After that, all animals were sacrificed for further study.

LPS acute model: Male C57BL/6J mice (12-week) were randomly divided into four groups: saline, LPS, and LPS plus tenuigenin (25 mg/kg) and LPS plus tenuigenin (50 mg/kg). To induce NLRP3 activation and IL-1β secretion, mice were injected intraperitoneally with LPS (20 mg/kg) alone and LPS plus tenuigenin (25 mg/kg or 50 mg/kg). After 6 h, the serum samples were collected and the IL-1β level was measured by ELISA. Animals were sacrificed, and brains were harvested.

### Open field test

Spontaneous locomotor activity was assessed using the open field test in a Tru Scan 2.0 system (Coulbourn Instruments, Allentown, PA, USA). Locomotor activity was assessed in automated activity chambers connected to a digital scan analyzer that transmitted the number of infrared beam breaks (activity data) to the instrument. Total movement distance (cm) was recorded across a 60-min recording period.

### Rotarod test

An accelerating rotarod was used to evaluate motor coordination and balance. Mice were placed on a rotating rod (Rota Rod Rotamex 5, Columbus Instruments, USA) at a speed of 5 rounds per minute. The speed of the rotarod accelerated to 40 rounds per minute. The latency time (sec) falling from the rod was automatically recorded. Each mouse was given three trials, and the latency times were averaged.

### High-performance liquid chromatography (HPLC)

The dopamine (DA) contents, and its metabolites dihydroxyphenylacetic acid (DOPAC) and homovanillic acid (HVA) in striatum, were determined using an HPLC apparatus with an electrochemical detector (Model 5600A CoulArray Detector System ESA, MA, USA). Tissues were homogenized in 200 mM ice-cold perchloric acid and the homogenate placed in an ice bath for 60 min. The sample was then centrifuged at 15,000*g* for 20 min at 4 °C, and the supernatant was transferred to a clean tube. A one-half volume of the solution containing 20 mM potassium citrate, 300 mM potassium dihydrogen phosphate, and 2 mM Na_2_EDTA was added and mixed thoroughly to precipitate the perchloric acid. After incubating in an ice bath for 60 min, the mixture was centrifuged at 15,000*g* for 20 min at 4 °C. The supernatant was filtered through a 0.22-μm filter and injected into the HPLC system. The mobile phase was 125 mM sodium citrate buffer supplemented with 20% methanol, 0.1 mM Na_2_EDTA, and 0.5 mM 1-octanesulfonic acid sodium salt. The flow rate was set at 1.2 mL/min.

### Immunohistochemical studies and quantitative evaluation

Brain samples were collected and postfixed in 4% PFA at 4 °C overnight. They were transferred to 15% sucrose in phosphate-buffered saline (PBS) overnight and then to 30% sucrose overnight till the brain sunk to the bottom of the tube. Coronal sections (30 μm) were cut by a freezing microtome (Leica, Germany) and stored in an antifreeze solution. Sections of substantia nigra were collected for immunohistochemistry according to a previous report [16]. Briefly, they were incubated overnight with the antibody against TH or Iba-1 overnight at 4 °C. After rinsing three times with PBS, sections were incubated with a second antibody (1:200) and AB work solution (Vector Laboratories, Burlingame, CA, USA) for 30 min at 37 °C. DAB solution was used to visualize the staining.

Images were observed, and photos were taken under a confocal microscope (Axiovert LSM510, Carl Zeiss Co., Germany). The immunostaining signals were quantitatively analyzed using the Optical Fractionator method with Microbrightfield Stereo-Investigator software (Stereo Investigator software, Microbrightfield, VT, USA). The total number of TH-IR neuron and Iba-1-IR microglia in the entire extent of SNc were counted. Briefly, the regions of SNc in the midbrain sections were outlined at low magnification (×40). For TH^+^ and Iba-1^+^ cells, the counting frame size was 50 μm × 50 μm and the sampling grid size was 100 μm × 100 μm. All stereological analyses were performed under the ×200 magnification. The sampling scheme was designed to have a coefficient of error < 10% in order to get reliable results. Each brain contained 6 serial sections at 6 intervals. For immunohistochemical staining, select one series of sections per mouse. The total numbers of immunoreactive cells in the entire extent of SNc were counted from 4 to 6 mouse brains per group.

### Cell culture and treatment

Murine BV-2 microglia cells were maintained in DMEM/F12 (1:1) media supplemented with 10% fetal bovine serum and antibiotics at 37 °C in a humidified incubator under 5% CO_2_. For inducing inflammasome activation, 1 × 10^6^ cells were plated in 6-well plate overnight and the medium were changed to opti-MEM in the following morning, and then, the cells were primed with LPS (500 ng/ml) for 3 h. After that, the cells were stimulated with 2.5 mM ATP for 30 min or 150 μg/ml monosodium urate crystals (MSU) for 4 h.

### Measurement of intracellular ROS formation

The ROS-specific fluorescent probe H2DCFDA was used to detect intracellular generation of ROS by modification. After drug treatment, BV2 cells were incubated with 10 μM DCFDA for 30 min. After washing 3 times with PBS, the intensity of fluorescence was determined by a multimode reader (Vario Skan Flash, 3001, Thermo Scientific) under an emission wavelength at 530 nm and excitation wavelength at 485 nm. The obtained values were presented as folds of the controls.

### Enzyme-linked Immunosorbent assay (ELISA)

Cells were treated with different stimuli. The concentration of IL-1β in the cell culture supernatant or serum was measured by mouse IL-1β ELISA Kit according to the manufacturer’s instructions.

### Purification of cell culture supernatant protein

The cell culture supernatant was collected and centrifuged to remove dead cells, and the supernatant was transferred into new tubes. Then, 500 μL methanol and 125 μL chloroform were added to precipitate supernatant, vortex, and centrifuge 16,000*g* for 5 min. The upper phase was discarded without touching the protein disk, and 500 μL methanol was added for washing and centrifuged at 16,000*g* for 5 min. The supernatant was removed, and the pellet was dried at 37 °C for 5 min. Ultimately, 50 μL 2.5 × loading buffer was added with DTT and vortex. The samples were boiled and loaded on 15% gels.

### Western blotting analysis

Tissues and cells protein lysates were quantified by Bradford assays (Bio-Rad, Hercules, CA, USA). Proteins were electrophoresed through a 8–15% SDS-polyacrylamide gel and blotted to PVDF membrane. Blots were probed with the following primary antibodies: anti-NLRP3 (1:1000), anti-caspase-1 (1:500), anti-IL-1β (1:800), and anti-β-actin (1:5000). The signal was visualized using an Odyssey Infrared Imaging System (LI-COR Biosciences, Lincoln, NE, USA) according to the manufacturer’s instructions. The signals were also monitored by the Odyssey IR imaging system.

### Statistical analysis

All values are expressed as the mean ± SEM and were analyzed by one-way ANOVA or Student’s *t* test as appropriate by using Prism 5.0 software (GraphPad Software, San Diego, CA). *P* < 0.05 was considered significant.

## Results

### Tenuigenin improves motor impairment and increases dopamine level in the striatum of MPTP PD mice

**Fig. 2:**
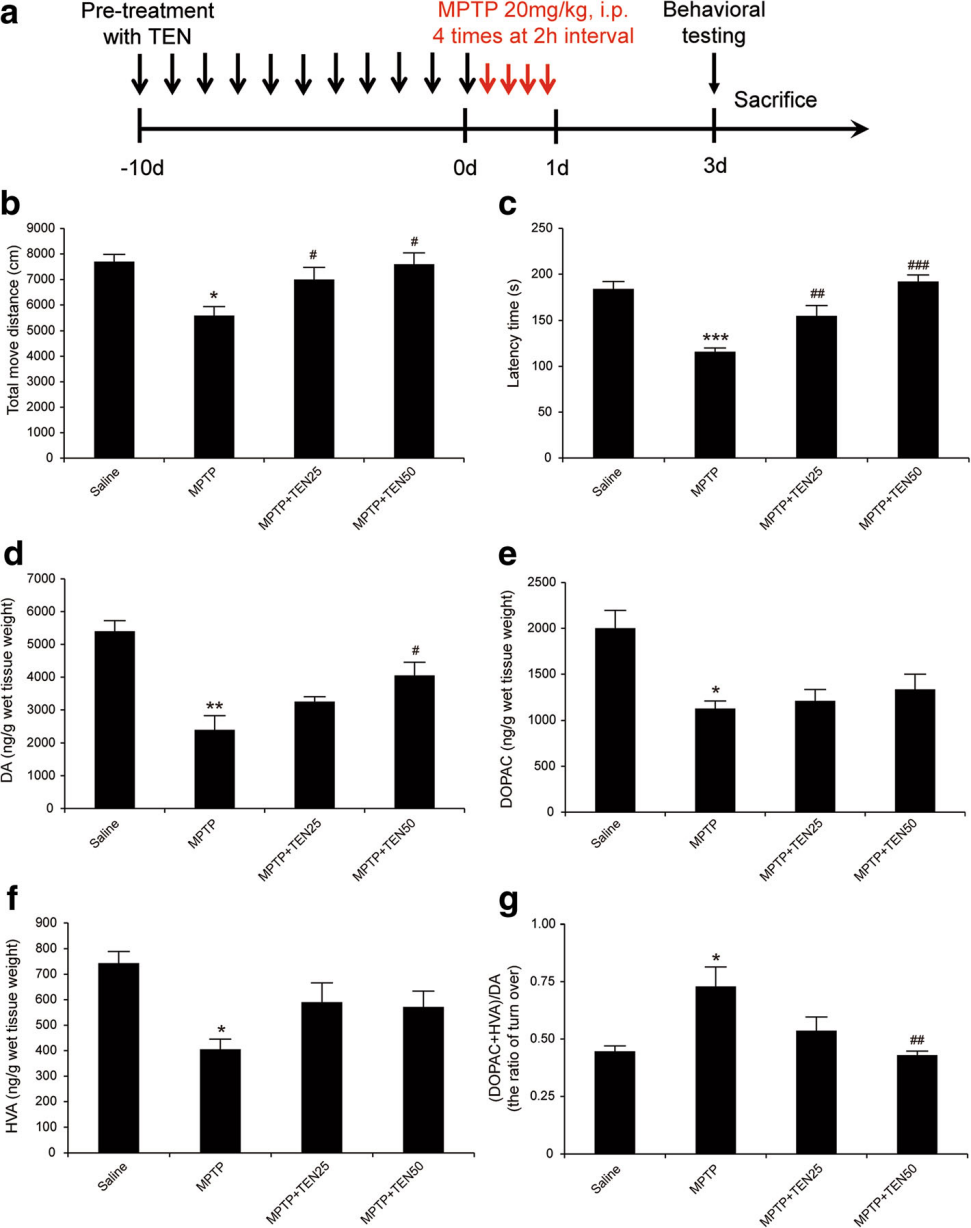
Tenuigenin improved motor behavior and increased dopamine levels in MPTP acute model mice. **a** Experimental procedure and drug administration schemes. Black and long arrows represented the administration of tenuigenin, while red and short arrows represented the administration of MPTP. **b** Effects of tenuigenin on total move distance in each group. Locomotor activity in 60 min was detected and analyzed by Truscan2.0 system 3 days after MPTP injections. **c** Effects of tenuigenin on the duration of mice stayed on the rotating rod. Latency time was detected 3 days after MPTP injections. **d** DA, **e** DOPAC, and **f** HVA were analyzed by HPLC. **g** The ratio of (DOPAC + HVA)/DA which represented the rate of DA metabolism. *n* = 12 for each group. Data are expressed as mean ± SEM, one-way ANOVA, *p < 0.05, **p < 0.01, and ***p < 0.001 vs. saline group. ^#^ *p* < 0.05, ^##^ *p* < 0.01, and ^###^ *p* < 0.001 vs. MPTP group

A classic systemic model is based on the administration of MPTP, with selective toxicity for dopaminergic neurons [17]. To explore the neuroprotective effects of tenuigenin in PD, we established a MPTP-induced acute model of PD in adult mice and determined whether tenuigenin improved MPTP-induced motor deficits. Experimental procedure and drug administration are shown in Fig. 2a. Open field test is a commonly used method to evaluate motor impairment. As shown in Fig. 2b, MPTP group was found to have a significant reduction in total movement distance compared to the saline group. MPTP mice pretreated with tenuigenin in both high dose (50 mg/kg) and low dose (25 mg/kg) exhibited an improvement in locomotor activity compared to MPTP group. Then, we used an accelerating rotarod test to determine motor coordination and balance. As shown in Fig. 2c, MPTP injections remarkably decreased the latency time compared with those in the saline treatment group. Moreover, high dose of tenuigenin displayed better effect on the improvement of motor behavior. These results indicate that tenuigenin administration recovered MPTP-induced motor impairment.

Furthermore, we detected the levels of DA and its metabolites, DOPAC and HVA, in the striatum by HPLC analysis. The results showed that these neurotransmitter substances in MPTP PD mice were significantly decreased by 44.4, 56.3, and 54.5%, respectively, compared with those in saline-treated mice. However, treatment of MPTP PD mice with 50 mg/kg tenuigenin increased DA level compared with untreated PD mice, while the levels of DOPAC and HVA did not significantly increase (Fig. 2d–f). Then, we calculated the ratio of (DOPAC + HVA)/DA which represented the rate of DA metabolism. As shown in Fig. 2g, high dose of tenuigenin decreased the rate of DA metabolism accelerating by MPTP. These results suggest that tenuigenin inhibited DA metabolism and elevated DA level in the striatum of MPTP PD mice.

### Tenuigenin protects dopaminergic neurons against MPTP-induced degeneration and suppresses NLRP3 inflammasome activation in substantia nigra

**Fig. 3:**
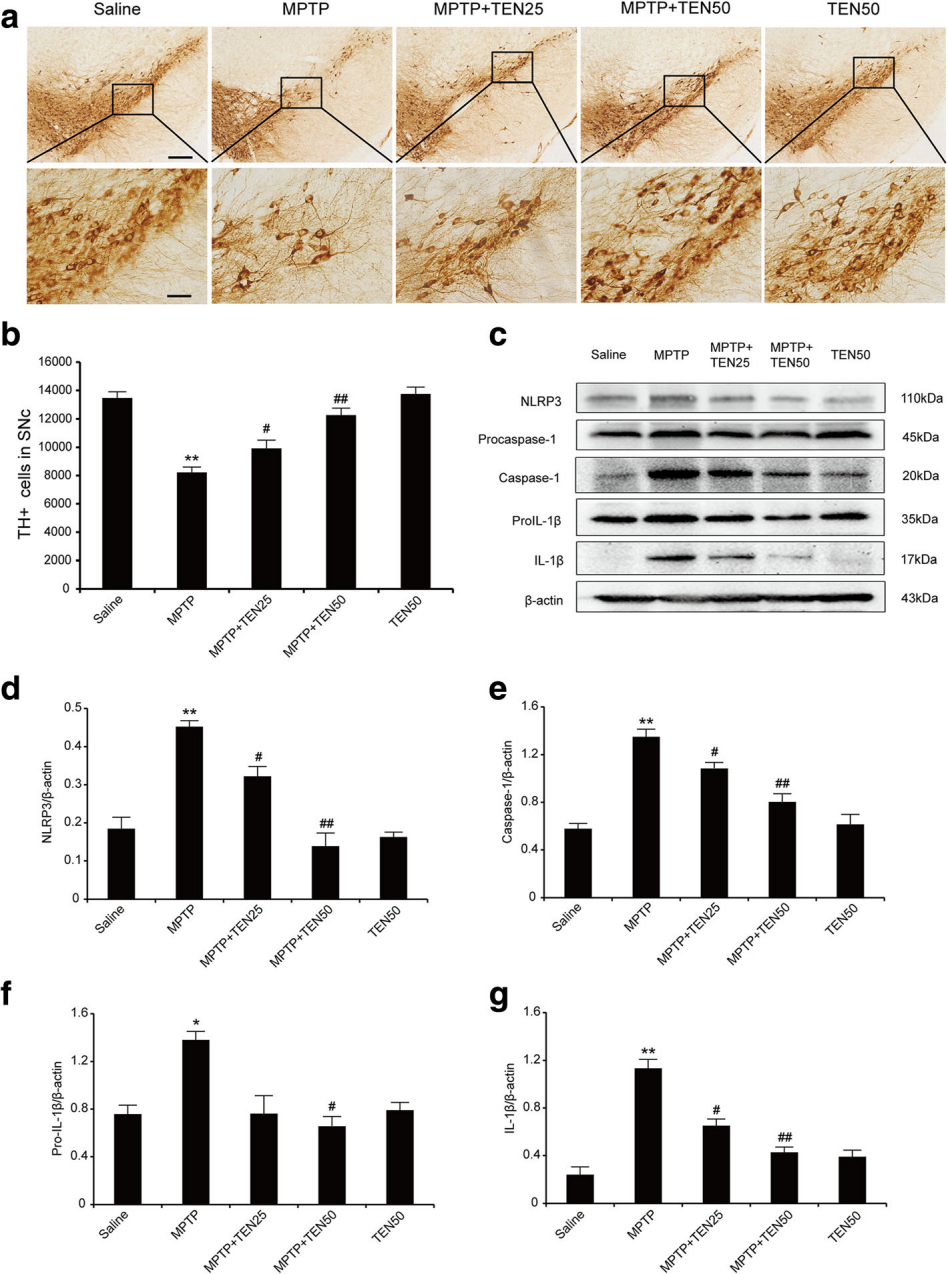
Tenuigenin prevented dopaminergic neuron degeneration and NLRP3 inflammasome activation induced by MPTP in the SNc of mice. **a** Immunohistochemical staining of TH-positive neurons in the SNc of each group. **b** Stereological counts of TH-positive cells in the SNc of each group. **c**–**g** Immunoblot analysis of NLRP3, pro-IL-1β, pro-caspase-1, cleaved caspase-1 (p20), and IL-1β in the substantia nigra of each group. *n* = 4 for each group. Data are expressed as mean ± SEM, one-way ANOVA, *p < 0.05 and **p < 0.01 vs. saline group. ^#^ *p* < 0.05 and ^##^ *p* < 0.01 vs. MPTP group. Scale bar represents 200 μm (upper) or 50 μm (under)

To demonstrate whether tenuigenin protects dopaminergic neurons from MPTP damage, we detected tyrosine hydroxylase in SNc by immunohistochemistry. As shown in Fig. 3a, administration of MPTP resulted in a 38.9% loss of TH^+^ neurons in SNc compared with those in saline-treated mice. Interestingly, high dose (50 mg/kg) of tenuigenin significantly increased the number of TH^+^ neurons by 49.2% in the SNc of MPTP PD mice, while low dose (25 mg/kg) of tenuigenin increased by 20.6%. These results indicate that tenuigenin exerts a beneficial effect on dopaminergic neuronal degeneration.

Activation of microglia in both striatum and substantia nigra had been well documented to occur in the MPTP model of PD [2, 18]. Our previous studies had reported the NLRP3 inflammasome was activated in the substantia nigra of MPTP mice [11]. So we examined whether tenuigenin had an impact on the activation of NLRP3 inflammasome. As shown in Fig. 3c–g, MPTP treatment significantly evaluated the levels of inflammasome including NLRP3, caspase-1, pro-IL-1β, and IL-1β, while tenuigenin suppressed the activation of NLRP3 inflammasome in substantia nigra, especially with the high dose of tenuigenin. These date imply that tenuigenin inhibits the activation of NLRP3 inflammasome induced by MPTP in substantia nigra.

### Tenuigenin inhibits NLRP3 inflammasome activation in BV2 microglia cells

**Fig. 4:**
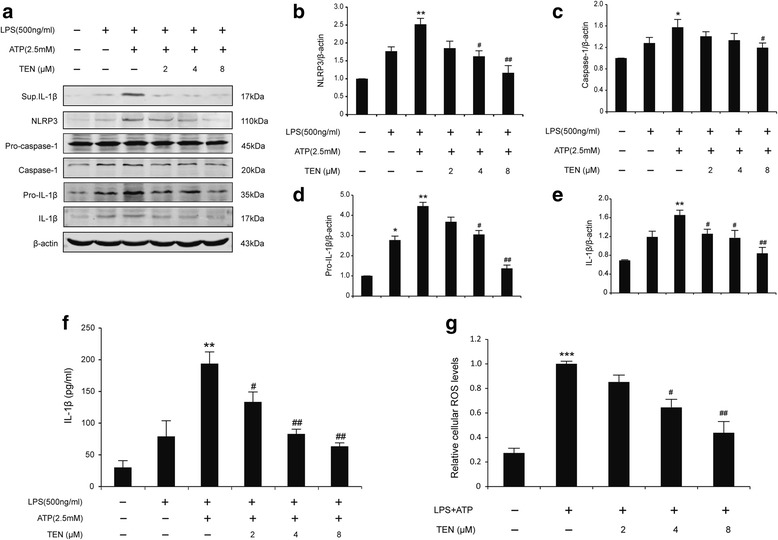
Tenuigenin inhibits NLRP3 inflammasome activation induced by ATP in BV2 microglia. **a**–**e** Immunoblot analysis of IL-1β in culture supernatants of LPS-primed BV2 treated for 3 h with various doses of tenuigenin and then stimulated with ATP (2.5 mM) for 30 min, and immunoblot analysis of NLRP3, the precursors of IL-1β (pro-IL-1β) and caspase-1 (pro-caspase-1), cleaved caspase-1 (p20), and IL-1β in lysates of those cells. **f** ELISA of matured IL-1β in supernatants from LPS-primed BV2 treated for 3 h with various doses of tenuigenin and then stimulated with ATP. **g** Cells were stained with DCFDA labeling and the quantitative analysis of ROS levels. All of the data were from the three independent experiments. Data are expressed as mean ± SEM, one-way ANOVA, *p < 0.05, **p < 0.01 and ***p < 0.001 in comparison with untreated control. ^#^ *p* < 0.05 and ^##^ *p* < 0.01 in comparison with LPS+ATP group

Then, we sought to determine whether tenuigenin has an effect on NLRP3 inflammasome activation in vitro. We used an immortalized murine microglial cell line, BV2, because it is an ideal alternative model system for primary microglia cultures [19]. The NLRP3 inflammasome is activated by endogenous stress-associated danger signals, such as ATP, nigericin, and MSU [9]. Since inflammasome activation is secretion of the cleaved bioactive form of IL-1β, researchers working in the inflammasome field typically “prime” the cells with LPS before performing the actual stimulation of the inflammasome with activators. Therefore, LPS-primed BV2 cells were pre-treated with tenuigenin before ATP challenge in the presence or absence of tenuigenin for 24 h. The results showed ATP induced caspase-1 activation and increased IL-1β production in BV2 microglia. The expression levels of those components of inflammasome, such as NLRP3, caspase-1, pro-IL-1β, and IL-1β, were downregulated by tenuigenin in a concentration-dependent manner (Fig. 4a–e). In particular, the inhibition of IL-1β secretion in cell supernatant also followed in a dose-dependent manner by Western blot and ELISA analysis (Fig. 4f). These data indicate that tenuigenin indeed can suppress NLRP3 inflammasome activation in microglia. To dissect the underlying molecular mechanisms, we measured the ROS production, which is believed to be a common NLRP3 activator. As shown in Fig. 4g, LPS-primed BV2 cells were pretreated with tenuigenin in different concentration before ATP challenge. High dose (8 μM) of tenuigenin remarkably decreased ATP-induced ROS production as detected by the fluorescence of H2DCFDA in BV2 microglia.

**Fig. 5:**
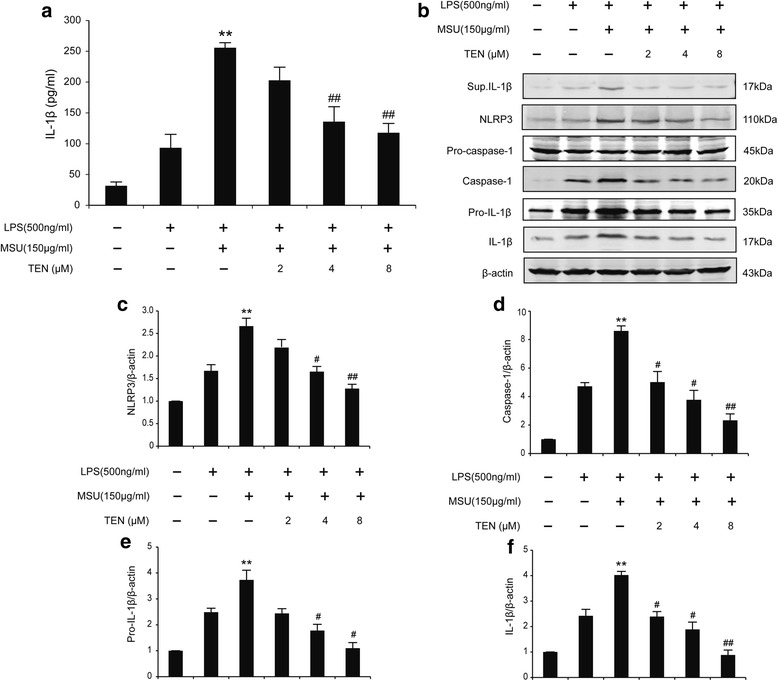
Tenuigenin inhibits NLRP3 inflammasome activation induced by MSU in BV2 microglia. **a** ELISA of IL-1β in supernatants from LPS-primed BV2 treated for 3 h with various doses of tenuigenin and then stimulated with MSU. **b**–**f** Immunoblot analysis of IL-1β in culture supernatants of LPS-primed BV2 treated for 3 h with various doses of tenuigenin and then stimulated with MSU (150 μg/ml) for 4 h, and immunoblot analysis of NLRP3, pro-IL-1β, pro-caspase-1, cleaved caspase-1 (p20), and IL-1β in lysates of those cells. All of the data were from the three independent experiments. Data are expressed as mean ± SEM, one-way ANOVA, **p < 0.01 in comparison with untreated control. ^#^ *p* < 0.05 and ^##^ *p* < 0.01 in comparison with LPS+MSU group

In order to find whether tenuigenin only affect ATP-induced NLRP3 inflammasome activation, we examined another NLRP3 agonists, MSU. As shown in Fig. 5a–f, tenuigenin inhibited caspase-1 cleavage and IL-1β secretion induced by MSU, coincident with the result induced by ATP. The observed inhibitory effects of tenuigenin on NLRP3 inflammasome activation were also confirmed. These results suggest that tenuigenin is a potent and broad inhibitor for NLRP3 inflammasome activation.

### Tenuigenin attenuates microglia activation induced by LPS via suppressing NLRP3 inflammasome in substantia nigra

**Fig. 6:**
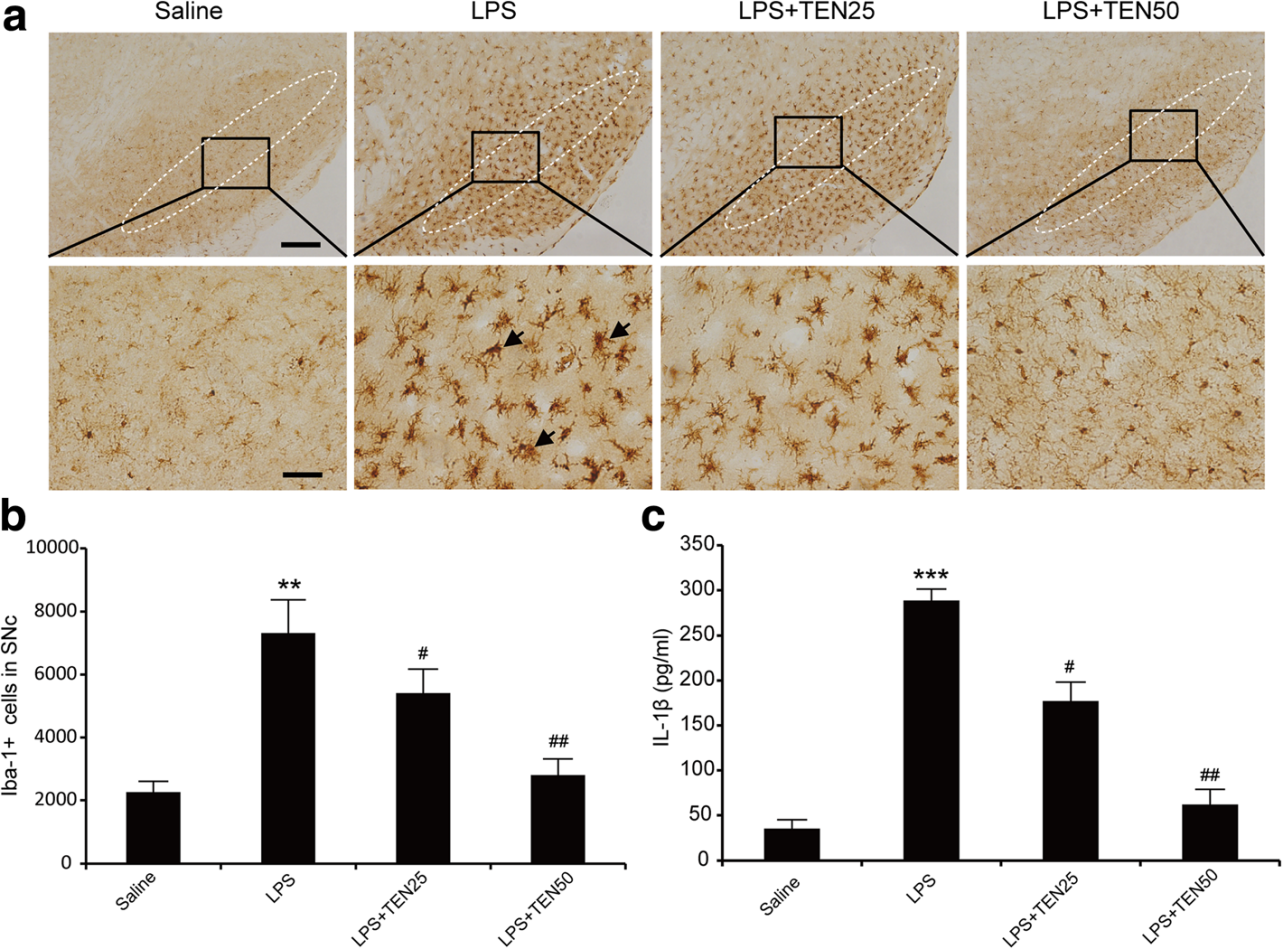
Tenuigenin attenuates microglia activation induced by LPS in SNc and suppress LPS-induced serum IL-1β production. **a** Immunostaining with Iba-1 to visualize the activation of microglia in the SNc of each group. Scale bar represents 200 μm (upper) or 50 μm (under). **b** Stereological counts of Iba-1-positive cells in the SNc of each group. **c** ELISA of IL-1β in serum of each group. *n* = 6 for each group. Data are expressed as mean ± SEM, one-way ANOVA, **p < 0.01 and ***p < 0.001 vs. saline group. ^#^ *p* < 0.05 and ^##^ *p* < 0.01 vs. LPS group

**Fig. 7:**
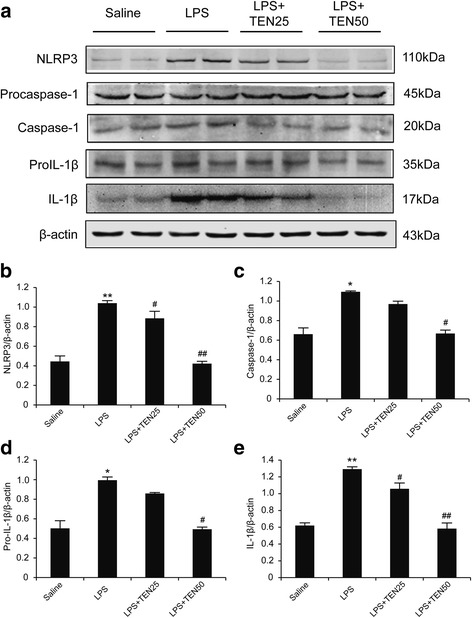
Tenuigenin suppressed NLRP3 inflammasome activation induced by LPS in SNc of mice. **a**–**e** Immunoblot analysis of NLRP3, pro-IL-1β, pro-caspase-1, cleaved caspase-1 (p20), and IL-1β in the SNc of each group. *n* = 6 for each group. Data are expressed as mean ± SEM, one-way ANOVA, *p < 0.05 and **p < 0.01 vs. saline group. ^#^ *p* < 0.05 and ^##^ *p* < 0.01 vs. LPS group

LPS, the principal component of the outer membrane of Gram-negative bacteria, is used to raise pro-IL-1β levels prior to the NLRP3 inflammasome stimulation. Peripheral administration of LPS alone could activate microglia in the brain and lead to inflammasome activation in vivo [20, 21]. Therefore, we detected whether tenuigenin can inhibit the activation of microglia and NLRP3 inflammasome in vivo by the treatment of LPS. Mice were injected intraperitoneally with LPS in the presence or absence of tenuigenin. After 6 h, the serum samples and brains were harvested. We performed immunohistochemistry with the classic antibody specific for Iba-1 to assess morphological microglia activation in SNc. The results showed that a large number of activated microglia with enlarged cell bodies (arrows shown in Fig. 6a) were observed in the SNc of LPS-treated mice compared with the saline group, while tenuigenin administration dramatically suppressed the activation of microglia in SNc (Fig. 6a, b). Furthermore, tenuigenin treatment significantly decreased serum IL-1β production ascending by LPS (Fig. 6c). Then, we wanted to know whether tenuigenin can prevent NLRP3 inflammasome activation induced by LPS in vivo. To address this question, we precisely isolated the tissues of ventral midbrain in each group. Western blotting analysis revealed that LPS significantly increased NLRP3, caspase-1, pro-IL-1β, and IL-1β expression, and this effect was reversed by tenuigenin treatment especially in the high-dose group (Fig. 7a–e). These results suggest that tenuigenin can inhibit the NLRP3 inflammasome activation in vivo.

## Discussion

In the present study, we demonstrated tenuigenin, a major active component of *Polygala tenuifolia* root, exerted the neuroprotective effects on dopaminergic neurons in MPTP mouse model of PD. We further found that tenuigenin inhibits the NLRP3 inflammasome activation both in vitro and in vivo. Our findings indicate that tenuigenin may target the NLRP3 inflammasome in microglia to attenuate MPTP-triggered neuronal degeneration, suggesting the potential of tenuigenin in prospective therapy for PD.

Our previous studies had demonstrated that tenuigenin exhibited the neuroprotective effects in neurodegenerative diseases [14, 15]. Nevertheless, it remains unclear whether tenuigenin plays a crucial role in animal models of PD. MPTP mouse model remains the most commonly used animal model of PD, which aims to reproduce the pathological and behavioral changes of the human disease [17]. In this study, we used the MPTP acute mouse model to evaluate the effect of tenuigenin on the protection of dopaminergic neurons. Because tenuigenin can pass through the blood-brain barrier easily due to its lipophilic characteristics and small molecular size, we pre-treated the drug for 10 days by intraperitoneal injection before administration with MPTP. The data showed tenuigenin improved MPTP-induced motor deficits and elevated DA level in the striatum. Also, tenuigenin markedly ameliorated the degeneration of dopaminergic neurons in SNc and decreased the expression of NLRP3 inflammasome components. The results confirmed the therapeutic effect of tenuigenin on MPTP mouse model of PD and showed the anti-inflammatory role of tenuigenin may target with NLRP3 inflammasome.

Increasing evidence including our previous studies had already shown that the NLRP3 inflammasome is involved in the progression of PD [10, 11, 22, 23]. NLRP3 inflammasome-mediated IL-1β production requires two signals. The first signal induces nuclear transcription factor-κB (NF-κB) to increase the expression of NLRP3 and proIL-1β, which is a prerequisite for inflammasome activation. The second signal directly activates the NLRP3 inflammasome to induce caspase-1 cleavage, leading to the maturation of IL-1β. NLRP3 protein expression levels have been shown to be a limiting step in inflammasome activation [24, 25]. In order to confirm the effect of tenuigenin on NLRP3 inflammasome, we activated the NLRP3 inflammasome in BV-2 microglial cells by ATP or MSU, which are the activators of NLRP3 inflammasome. Here, we found that tenuigenin reduced the expression of NLRP3 and pro-IL-1β in BV2 microglia. We also observed that tenuigenin suppressed activation of caspase-1 and the maturation of IL-1β in response to NLRP3 activators including ATP and MSU. These data indicate that tenuigenin inhibits both the priming (signal 1) and the activation (signal 2) of the NLRP3 inflammasome. NLRP3 was originally hypothesized to be a cytosolic receptor, with such a broad range of stimuli demonstrated to cause its activation. ROS, produced by many known activators of NLRP3 inflammasome, are shown to be a critical mechanism triggering NLRP3 inflammasome formation and activation [26, 27]. In this study, we found that tenuigenin decreased the ROS production induced by LPS and ATP in BV2 microglia. These data demonstrate that tenuigenin inhibits the activation of NLRP3 inflammasome through controlling the production of ROS.

Microglia activation has been known to play an important role in neuroinflammation of PD. Activated microglia release various pro-inflammatory and cytotoxic factors, such as IL-1β, IL-6, TNF-α, and ROS. The accumulation of these factors is considered to contribute to the progressive loss of dopaminergic neurons. The pharmacologic regulation of microglial activation could protect dopaminergic neurons from inflammatory injury [28, 29]. Peripheral administration of LPS could activate microglia in the brain and lead to inflammasome activation which might through the indirect way (e.g., by inducing the release of endogenous NLRP3 activators such as ATP or uric acid) [21, 30]. Here, we activated microglia and NLRP3 inflammasome through acute treatment with LPS by intraperitoneal injection. Iba-1 immunohistochemistry showed a large number of activated microglia cells were observed in the SNc of LPS-treated mice. Tenuigenin administration remarkably suppressed the activation of microglia in the brain and decreased serum IL-1β production increasing by LPS. Furthermore, tenuigenin treatment abolished LPS-induced activation of NLRP3 inflammasome in substantia nigra. These results suggest that tenuigenin alleviates microglia activation in SNc and inhibits NLRP3 inflammasome activation in vivo.

## Conclusions

**Fig. 8:**
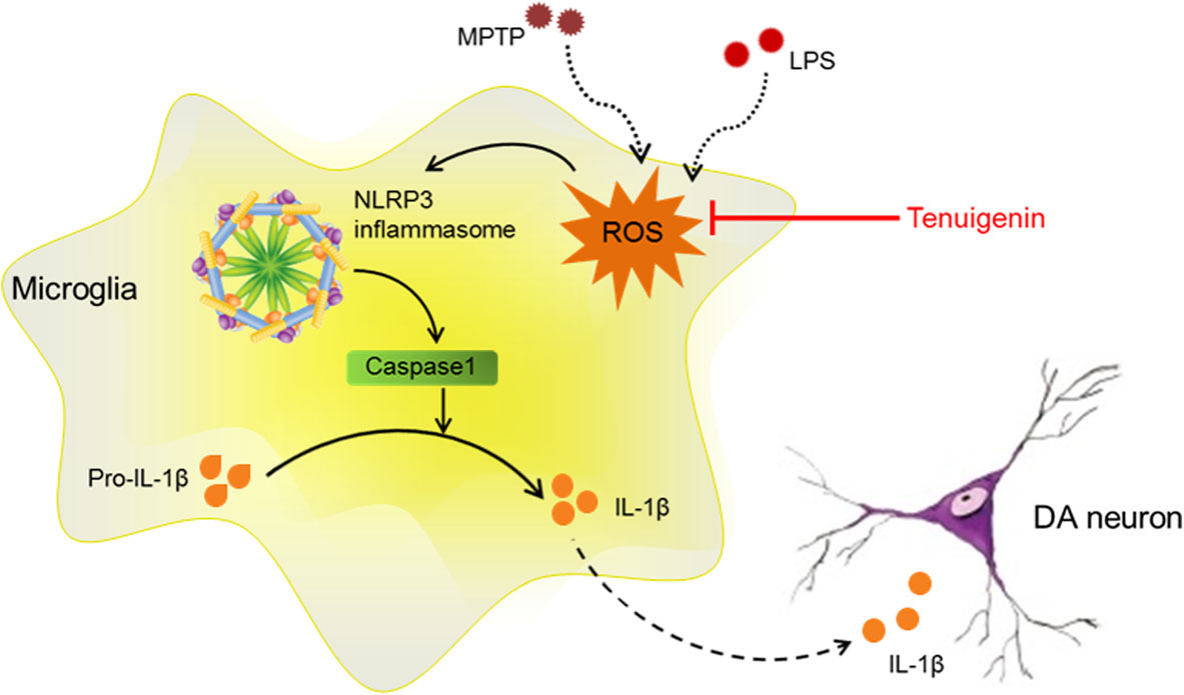
Schematic illustration demonstrates that tenuigenin protects dopaminergic neurons from inflammation via controlling the production of ROS and inhibiting NLRP3 inflammasome in microglia

Our results demonstrate that tenuigenin exerts the neuroprotective effects on dopaminergic neuronal degeneration and motor deficits in MPTP PD mice. Our results also demonstrate that tenuigenin can prevent neurotoxin-induced neuroinflammation via controlling the production of ROS and inhibiting NLRP3 inflammasome in microglia (Fig. 8). Collectively, our findings reveal that tenuigenin confers an anti-inflammation effect partly through inhibiting NLRP3 inflammasome activation, and suggest the promising clinical use of tenuigenin in NLRP3 inflammasome driven inflammatory diseases such as PD.

## Abbreviations

6-OHDA: 6-Hydroxydopamine
ANOVA: Analysis of variance
ATP: Adenosine 5′-triphosphate
BBB: Blood-brain barrier
COX-2: Cyclooxygenase-2
DA: Dopamine
DAMPs: Danger-associated molecular patterns
DOPAC: Dihydroxyphenylacetic acid
ELISA: Enzyme-linked immunosorbent assay
H2DCFDA: 2′, 7′-Dichlorodihydrofluorescein diacetate
HPLC: High-performance liquid chromatography
HVA: Homovanillic acid
Iba-1: Ionized calcium-binding adaptor molecule 1
IL-1β: Interleukin-1β
iNOS: Inducible nitric oxide synthase
LPS: Lipopolysaccharide
MPTP: 1-Methyl-4-phenyl-1,2,3,6-tetrahydropyridine
MSU: Monosodium urate crystals
NF-κB: Nuclear transcription factor-κB
NLRP3: Nod-like receptor family, pyrin domain-containing 3
NSAIDs: Nonsteroidal anti-inflammatory drugs
PAMPs: Pathogen-associated molecular patterns
PBS: Phosphate-buffered saline
PD: Parkinson’s disease
PGE2: Prostaglandin E2
ROS: Reactive oxygen species
SNc: Substantia nigra compacta
TEN: Tenuigenin
TH: Tyrosine hydroxylase
TNF-α: Tumor necrosis factor-α
